# *VERNALIZATION2* alters early tiller development in a facultative spring hexaploid bread wheat

**DOI:** 10.1111/nph.70907

**Published:** 2026-02-05

**Authors:** Dominique Hirsz, Harry Taylor, India Lacey, Wenxue Wu, Adam Gauley, Laura Dixon

**Affiliations:** ^1^ Leibniz Institute of Plant Genetics and Crop Plant Research (IPK) 06466 Gatersleben Germany; ^2^ Faculty of Biological Sciences University of Leeds Woodhouse Lane Leeds LS2 9NL UK; ^3^ Department of Plant Sciences University of Cambridge Downing Street Cambridge CB2 3EA UK; ^4^ State Key Laboratory of Crop Gene Resources and Breeding/Institute of Crop Sciences Chinese Academy of Agricultural Sciences Beijing 100081 China; ^5^ Agri‐Food and Biosciences Institute Newforge Lane Belfast BT9 5PX UK

**Keywords:** photoperiod, temperature, *Triticum aestivum*, vernalization, *VRN1*, *VRN2*, *ZCCT1*, *ZCCT2*

## Abstract

- An extended period of cold exposure enables the process of vernalization in winter cereals and is important for the synchronised timing of the floral transition. The cereal‐specific floral repressor *VERNALIZATION2* (*VRN2*) has an integral role in vernalization, yet this locus remains poorly characterised in facultative spring hexaploid wheat, *Triticum aestivum*.
- Through the generation of defined germplasm combined with bespoke experimental protocols, which enable a realistic simulation of annual field‐based UK growth conditions, we were able to distinguish gene expression and phenotypic differences at the subgenomic level of *VRN2* in hexaploid bread wheat.
- Our research in a facultative wheat suggests that the tandemly duplicated genes comprising the *VRN2* locus, *ZCCT1* and *ZCCT2*, have gene expression patterns that respond to multiple environmental factors. These genes also show coregulation, forming a regulatory loop between *ZCCT‐D1* and *ZCCT‐D2*. The function of these genes beyond the classic vernalization response is explored in a facultative wheat. Here, we identified that *VRN‐D2* regulates early tiller development, with an accelerated rate of secondary tiller emergence and presence of coleoptile tillers.
- The findings identify that the *VRN2* loci in bread wheat are formed of multiple genes, which have not only overlapping but also unique regulation and function. Selecting these genes individually may offer a route to alter wheat plant architecture without directly impacting vernalization requirement.

## Introduction

Bread wheat (*Triticum aestivum*) is the most widely cultivated cereal crop, made possible by allelic variations in several key genes, which have been enriched for during selection and breeding. However, bread wheat remains extremely vulnerable to the effects of climate change. Each global temperature increase of 1°C is expected to result in global losses in yield of 6%, with anticipated losses ranging from *c*. 4–20% for different regions (Asseng *et al*., 2015; Liu *et al*., 2016). The ability to improve and modify the adaptability of wheat to changing climate conditions is vital to maintain yield potential and support a rapidly increasing global population and food demand (Godfray *et al*., 2010).

One aspect of adaptability that impacts yield potential is the vernalization requirement. This is the requirement for a prolonged exposure to cold temperatures, coupled with short‐day photoperiods, which provides plants with a means for monitoring winter and timing reproductive development to favourable conditions. Vernalization is quantitative, so the length of exposure to cold temperatures directly determines the rate of floral transition. Flowering time is accelerated following a longer cold duration up to a point, after which the vernalization requirement is satisfied and cold temperatures have limited or negative impact on flowering date (Dixon *et al*., 2019). In countries with a suitable climate pattern to meet the vernalization requirement, vernalization‐requiring (termed ‘winter’ wheat) has a higher yield potential than other wheat (Woods *et al*., 2016; Jacott & Boden, 2020). Furthermore, it provides a number of other agronomic benefits including establishment and ground cover over winter and competition against weeds such as blackgrass (Liang & Richards, 1994; Andrew *et al*., 2015; Sainju *et al*., 2022).

Wheat varieties that do not require vernalization are termed ‘spring’ varieties, avoiding the pathway through suppression of floral repressors or constitutive expression of floral activators (Trevaskis *et al*., 2007; Kippes *et al*., 2016; Dixon *et al*., 2019). A further group exists, which are facultative wheats; these do not require vernalization to flower, but flowering time is accelerated when they experience vernalization. Changing environmental patterns are altering previously established agricultural practices, with the optimal timing of crop planting becoming less predictable. For winter crops, the variable winter temperatures are making the timing for the completion of vernalization unpredictable. This presents two major challenges: too early completion causing a shift in meristem development and a reduction in plant biomass, or too late completion delaying flowering and, in extreme cases, not being satisfied before warmer spring conditions, and so a substantial reduction in final yield. Increasingly, facultative wheats are being used, which can balance these requirements.

**Fig. 1:**
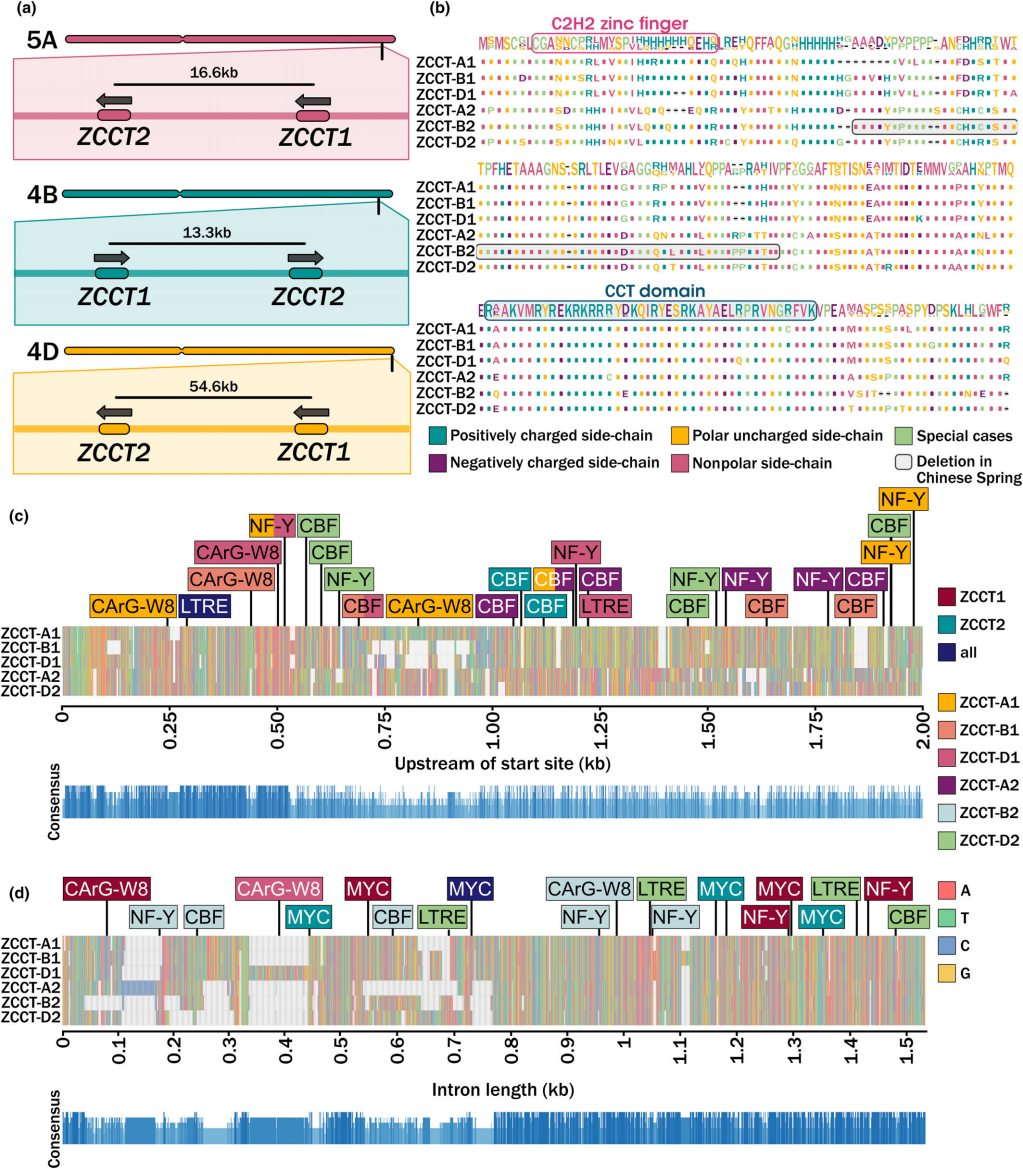
*VERNALIZATION2* (*VRN2*) location and sequence analysis. (a) A schematic showing the direction and locations of each *ZCCT1* and *ZCCT2* gene on each chromosome, and subgenome A, B and D in *Triticum aestivum* as measured from the midpoint of each gene. (b) The aligned protein sequences for each homoeologous copy of ZCCT1 and ZCCT2, highlighting the putative C_2_H_2_ zinc finger (pink) and CONSTANS, CONSTANS‐like and TOC1 (CCT) domain (blue). (c) A consensus sequence indicates the level of conservation across the five ZCCT genes annotated in the v1.2 reference cultivar ‘Chinese Spring’ for the promoter region, 2‐kb upstream of the start site. Motifs were identified using ‘PlantPAN 4.0’ (Chow *et al*., 2024). Nucleotides are indicated based on colour shown in the key and motifs of interest are indicated, with colours indicating whether the motif is conserved across all three homoeologous copies of ZCCT1 (maroon), ZCCT2 (teal) or across all ZCCT1 and ZCCT2 promoter regions (navy). Motifs that are specific to only one ZCCT promoter region are also indicated as indicated in the key. CArG‐W8, CArG box motif; CBF, C‐REPEAT BINDING FACTOR; LTRE, low temperature responsive element; NF‐Y, NUCLEAR FACTOR Y; A, adenine; T, thymine; C, cytosine; G, guanine. D as for C, but for the intron.

Understanding the contributions of the genetic components of the vernalization pathway is important to enable the development of wheat with a variety of vernalization responses. The genetic core of the vernalization pathway in cereals involves *VERNALISATION1* (*VRN1*), *VRN2* and *FLOWERING LOCUS T1* (*FT1*), also called *VRN3*. Mutations in the promoter or first intron of *VRN1* lead to overexpression, which results in a dominant spring wheat phenotype (Dixon *et al*., 2019). *VRN2* is a monocot‐specific floral repressor, highly expressed before vernalization and gradually repressed following cold exposure by the increasingly expressed *VRN1* (Distelfeld *et al*., 2009). Although *VRN2* is generally referred to as a gene, it is a locus made of two tandemly duplicated genes: *ZCCT1* and *ZCCT2* (Yan *et al*., 2004). It has a putative C_2_H_2_ zinc finger domain and a CCT domain, named after the genes *CONSTANS*, *CONSTANS‐like* and *TIMING OF CAB 1* identified in *Arabidopsis thaliana*. Notably, *VRN2* is part of a known translocation between chromosome 4A and 5A (Ma *et al*., 2013). Therefore, the *VRN2* gene in bread wheat refers to a total of six genes: *ZCCT‐A1* and *ZCCT‐A2* on chromosome 5A, *ZCCT‐B1* and *ZCCT‐B2* on 4B and *ZCCT‐D1* and *ZCCT‐D2* on 4D, all on the distal long arm of the chromosomes (Fig. 1a). The distance between these genes also differs between chromosome pairs, raising the possibility that *ZCCT* genes are under independent regulation and possibly even selection. It also highlights that the individual genes of the *VRN2* loci could be nonredundant with respect to the vernalization response.

The role of each *ZCCT* gene varies across different cereal species, with the diploid *Triticum monococcum* showing high expression of *ZCCT1* and low levels of expression of *ZCCT2*. In this wheat species, *zcct1* mutants exhibit a spring phenotype despite the presence of a functional *ZCCT2* gene, leading to the conclusion that *ZCCT1* is the primary functional copy (Yan *et al*., 2004). By contrast, the tetraploid wheat species *Triticum turgidum* shows higher expression of *ZCCT2* than *ZCCT1* (Distelfeld *et al*., 2009). This species was used to generate a synthetic hexaploid wheat with a triple knockout of *VRN2* (Kippes *et al*., 2016), in which the D‐genome was contributed by *Aegilops tauschii*. In this synthetic hexaploid, *ZCCT‐B2* was identified as the key copy giving *VRN2* its function as a floral repressor (Kippes *et al*., 2016). This triple‐null has a spring wheat phenotype, with significantly varying flowering times dependent on combinations of functional *VRN2* genome copies (Kippes *et al*., 2016). A recent study in barley has also shown that the *ZCCT* genes can show differential expression in response to vernalization (Montardit‐Tarda *et al*., 2025). Understanding the role of each *VRN2* gene in natural hexaploid wheat will be of great interest, as this suggests the potential to modulate the vernalization requirement of wheat through allelic and copy number variation of specific *VRN2* genes.

Alongside monitoring ambient temperature to direct floral transition, photoperiod monitoring is integral to ensure floral transition is correctly timed. In both barley and rice, *VRN2* expression is regulated by photoperiod in conjunction with temperature (Trevaskis *et al*., 2006; Turner *et al*., 2013). Photoperiod monitoring genes such as *PHOTOPERIOD‐1* (*Ppd‐1*) are part of a complex network of gene expression, which regulates the central floral integrator, *FT1*, to ensure floral transition occurs when conditions are ideal (Shaw *et al*., 2020). Many of the genes with a role in monitoring photoperiod, including *Ppd‐1* and the *CONSTANS* (*CO*) genes, also contain the highly conserved CCT domain (Li *et al*., 2011; Li & Xu, 2017; Zheng *et al*., 2017). In VRN2, mutations in the arginine amino acids at 16, 35 and 39 in the CCT domain significantly alter protein properties and can render the protein nonfunctional (Distelfeld *et al*., 2009).

We asked how vernalization genes respond in facultative spring wheat under different environmental conditions and whether plant development could be altered to improve winter traits without impacting the vernalization response. We identified that the expression of the *VRN2* loci is regulated by both temperature and photoperiod in bread wheat, and this expression alters under variable conditions. Therefore, specific genes within the *VRN2* loci can be targeted for improving aspects of crop performance. In particular, we identify that altered function of *ZCCT‐D1* and ‐*D2* regulates early tiller outgrowth without impacting final tiller number and flowering time. This was also recently identified in barley and has the potential to improve water, light and nutrient efficiency as well as improve overall soil health and structure (Liang & Richards, 1994; ter Steege *et al*., 2005; Sainju *et al*., 2022; Montardit‐Tarda *et al*., 2025).

## Materials and Methods

### Plant materials and growth conditions

All plants were germinated at room temperature (21°C) on Whatman filter paper with 5 ml ddH_2_O in a 9‐cm Petri dish and seedlings transferred to 24‐well seed trays (each cell 50 mm × 48 mm × 52 mm) containing John Innes Cereal Mix (Backhaus *et al*., 2022). The *Triticum aestivum* L. cultivars used were either from laboratory stocks (*cv*. Cadenza) or obtained from the Germplasm Resource Unit for all TILLING mutants (Rakszegi *et al*., 2010; Krasileva *et al*., 2017). Plants used for generation of *zcct‐d2_m1* and *zcct‐d1_m1* mutant lines were then grown under glasshouse conditions (16 h : 8 h, 21°C : 15°C, light : dark). All germplasms used and developed in this study are listed in Supporting Information Table S1.

The facultative spring wheat cultivar Cadenza was used for the 24‐h time‐course expression analysis. These plants were grown in Sanyo Plant Growth cabinets under the following temperature conditions with a constant 12‐hour photoperiod: 16°C during the light period/10°C during dark, 10°C during light/16°C during dark, and 10°C constant and 16°C constant. Plants were grown in these conditions for 3 wk, and then leaf tip tissue was sampled at 5‐h intervals across a 24‐h period, with tissue from two individual plants pooled for each of three biological replicates.

Growth conditions for the long‐term seasonal gene expression experiment were conducted in a Conviron gen 2000 growth chamber with conditions listed in Table S2. The facultative spring wheat cultivar Cadenza and mutant lines *zcct‐d2_m1* and *zcct‐d1_m1* were grown in these conditions. Leaf tissue samples were taken each week an hour after the relative ‘dawn’ and ‘dusk’ for each day depending on when light changes occurred.

Tiller emergence was recorded every day for the first 35 d of growth. Flowering time was defined as half‐ear emergence from the flag leaf, GS55 on the Waddingtons scale. Spikelet number was counted for the spike from the primary tiller for each plant.

### Generation of *zcct‐d2_m1* and *zcct‐d1_m1* mutant lines

The two mutant lines were in the hexaploid *cv*. Cadenza background: Cadenza0810 and Cadenza1436 (with single nucleotide polymorphisms (SNPs) in *ZCCT‐D2* and *ZCCT‐D1* respectively) (Krasileva *et al*., 2017) were twice backcrossed. Plants were genotyped using primers detailed in Table S3 at the SNP of interest via KASP assay as described in Dixon *et al*. (2018) to identify homozygous plants. Genomic DNA was extracted using the chloroform:isoamyl alcohol method adapted from Paterson *et al*. (1993).

PCR amplification was conducted to confirm homozygosity at the SNP of interest using Q5 DNA Polymerase (NEB) with reagent quantities and conditions following the manufacturer's protocol. Primers are listed in Table S3. The PCR was separated using gel electrophoresis and the fragment was extracted using the Monarch® DNA Gel Extraction Kit (NEB, Ipswich, MA, USA) according to the manufacturer's protocol. Samples were then sequenced by Eurofins Genomics and analysed to confirm the SNP.

### Analysis of gene expression

To measure the expression, plants were sampled as outlined see ‘Plant materials and growth conditions’ in the Materials and Methods section. All plant samples were flash‐frozen in liquid nitrogen and stored at −70°C. The tissue was lysed using the TissueLyserLT (Qiagen, Hilden, Germany) using 3‐mm steel ball bearings, and RNA was then extracted for all leaf tissue samples using the Spectrum™ Plant Total RNA Kit (Sigma‐Aldrich, St. Louis, MI, USA) or Monarch® Total RNA Miniprep kit (NEB, Ipswich, MA, USA) according to the relevant manufacturer's protocol. Synthesis of cDNA was conducted according to either Dixon *et al*. (2018) or using the reverse transcriptase UltraScript 2.0 (PCR Biosystems, London, UK) according to the manufacturer's protocol. The cDNA was then diluted (1 : 10) and quantitative real‐time reverse transcriptase PCR (RT‐qPCR) was conducted using GoTaq® qPCR Master Mix (Promega, Madison, WI, USA) according to the manufacturer. The primers used are listed in Table S3, and the thermal cycling conditions performed using the CFX96™ Thermal Cycler (Bio‐Rad, Watford, Hertfordshire, UK) and protocol described in Dixon *et al*. (2018). Expression was calculated relative to the housekeeping gene *TraesCS5A02G015600* (Borrill *et al*., 2016), using the formula 2ΔCT where ΔCT = [expression of Gene of Interest] – [expression of *TraesCS5A02G015600*].

### Promoter and intron motif analysis

Gene and 2‐kb putative promoter sequences were extracted from Ensembl plants following alignments using BLAST for the ‘10+ Genomes’ hexaploid wheat pangenome cultivars and wheat relatives (Walkowiak *et al*., 2020). Additional hexaploid wheat sequences were extracted from the pangenome by Jiao *et al*., 2025. Sequences were aligned against Chinese Spring v.2.1 using MAFFT to identify each individual *ZCCT* gene. Identification of potential binding motifs was then conducted using the PLACE (Plant *Cis*‐acting Regulatory DNA Elements) database (Higo *et al*., 1999).

## Results

### *ZCCT1* and *ZCCT2* , which form the *VRN2* loci, contain different regulatory domains

The number of genes forming the *VRN2* loci in hexaploid bread wheat was believed to be six, *ZCCT‐A1* and ‐*A2*, *‐B* and *‐D*. However, a blast analysis of the reference genome *cv*. Chinese Spring (v.2.1) identified only five genes, missing *ZCCT‐B2*. Specific gene search in the region of *ZCCT‐B2* identified a truncated version of *ZCCT‐B2* in the reference, containing a 62 amino acid deletion (Fig. 1b). This highlights that the different *ZCCT1* and −2 genes could be selected independently despite being physically close on the chromosome (Fig. 1a). Comparison of the coding region of these sequences (Fig. 1b) shows that the putative zinc finger domain has a high level of variation across the different *ZCCT* genes compared with the CCT domain, which is known to be well‐conserved (Distelfeld *et al*., 2009; Li *et al*., 2011). There is also strong conservation from amino acid residues 73–93, which is not part of either domain but is included in the deletion in *cv*. Chinese Spring in *ZCCT‐B2*.

Through analysis of publicly available wheat pangenomes (Walkowiak *et al*., 2020; Jiao *et al*., 2025), we identified two main allelic variants for *ZCCT‐A1*, *‐B1* and ‐*A2* (Tables S4 and S5). The *ZCCT‐D1* and *‐D2* genes were shown to be fully conserved across all but one of the 39 varieties analysed, and *ZCCT‐B2* was able to be identified in all genotypes, although it was not annotated for most varieties. Comparison with ancestral wheat sequences shows little variation across the *ZCCT* genes (Table S6).

To begin to investigate the possibility of independent selection of these genes, we classified the regulatory domains within the intron and 2‐kb upstream of the putative start site for each of the *ZCCT* genes (Fig. 1c,d). Here, we could identify not only that the two genes *ZCCT‐1* and *‐2* contained unique regulatory domains but also that even within the *ZCCT‐1* or *‐2* genes, there is variation between the promoter regions. For example, in the 2‐kb upstream of the putative start site, a 0.25‐kb region was deleted in *ZCCT‐B1* and *‐D1* compared with *ZCCT‐A1*. To identify potential binding motifs, a database of known motifs across multiple vascular plant species was used (Higo *et al*., 1999). There was variation between genes for key motifs such as the CArG box, a binding site for MADS box transcription factors such as VRN1 (Deng *et al*., 2015; Chow *et al*., 2024), which was present in all three homoeologous copies of *ZCCT1* in different locations but not *ZCCT2* (Fig. 1c). Comparisons of the promoter regions for the main allelic variants of each *ZCCT* gene show some variation (*c*. 20–50 SNPs), which altered few key motifs (Dataset S1). *ZCCT‐B1* had the largest difference between alleles, showing low conservation *c*. 1.6‐kb upstream of the putative start site (Dataset S1). The ancestral wheats analysed also showed generally little variation between their promoters and the hexaploid wheat promoter, often similar (*c*. 20–50 SNPs) to the variation between allelic variants for *ZCCT‐A1*, *‐B1* and *‐A2* genes in hexaploid wheat. The analysis of the single intron was conducted in *cv*. Cadenza to include *ZCCT‐B2* and showed conservation was better in the latter half of the intron (*c*. 0.8–1.5 kb) with large deletions present in *ZCCT‐A2* and *ZCCT‐B2* and insertions present in *ZCCT‐D1* and *ZCCT‐A2*, as well as several deletions in all three homoeologous copies of *ZCCT2* compared with *ZCCT1* (Fig. 1d). This highlighted that the two *ZCCT* genes have diverged in regulatory domains and strongly suggested *ZCCT1* and *ZCCT2* are not functionally redundant and may be differentially regulated in response to environmental conditions.

### Core vernalization genes' expression alters depending on temperature and photoperiod conditions in facultative wheat

The domain analysis suggested that the *VRN2* genes could be under different regulation and so we hypothesised that some of the *VRN2* genes may offer a route to coordinate environmental responses and development without always impacting the vernalization response. It has already been established that expression of the *VRN2* genes is responsive to changes in photoperiod (Dubcovsky *et al*., 2006), so to investigate the effect of temperature, we first characterised how vernalization genes behave in the facultative spring wheat *cv*. Cadenza via RT‐qPCR analysis on 3‐wk‐old leaf tissue across a series of simulated environmental scenarios, all with the same day‐neutral (DN; 12 h : 12 h, light : dark) photoperiods. Alleles of the key vernalization genes for *cv*. Cadenza are shown in Table S7.

**Fig. 2:**
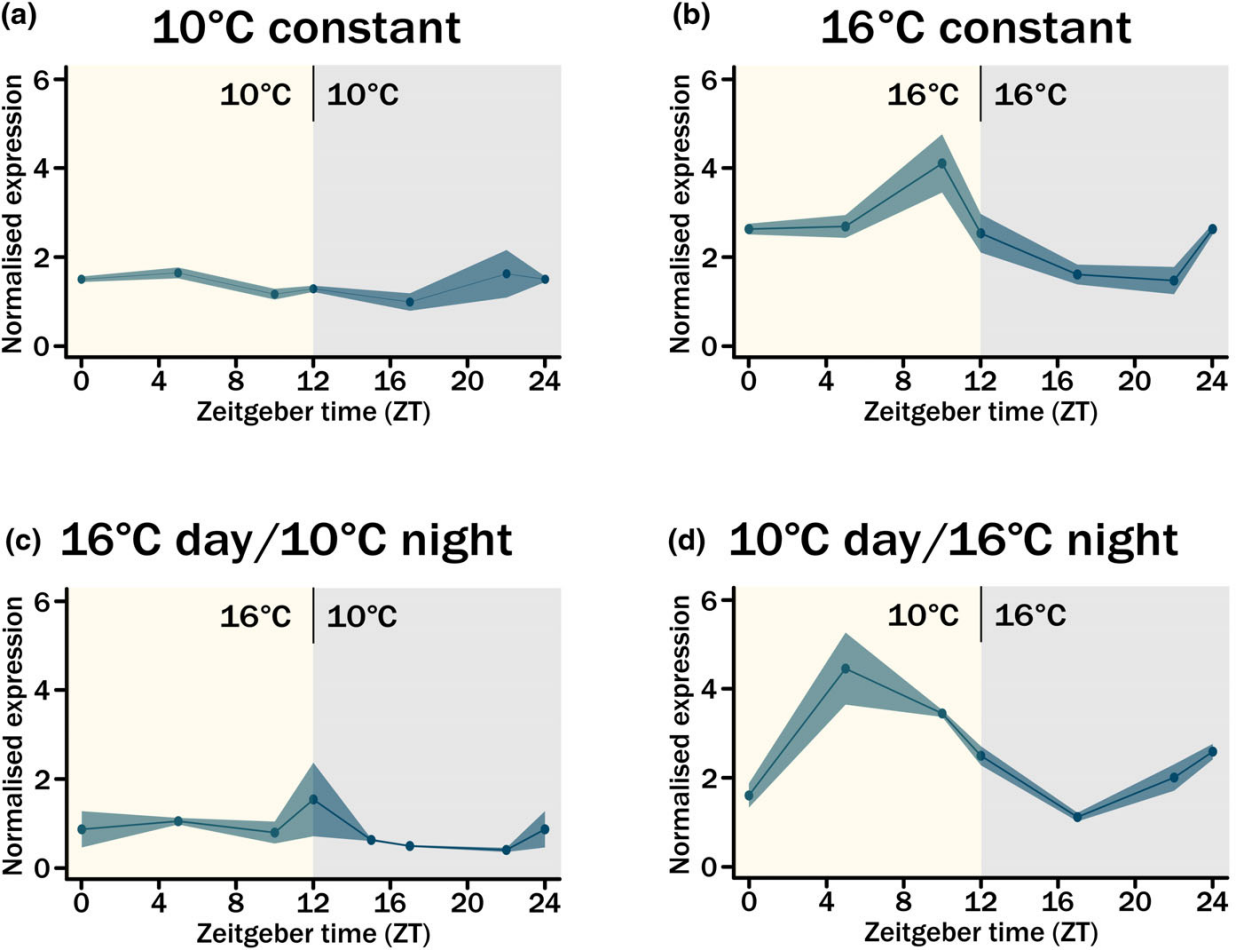
Expression of *VERNALIZATION1* (*VRN1*) under day‐neutral (DN) conditions with variable temperatures. A ribbon plot showing expression of *VRN1* for all three subgenome copies (A, B, D) in the facultative spring *Triticum aestivum cv*. Cadenza across a 24‐h time course. Leaf tissue was sampled after 3 wk of growth in 12‐h DN conditions under different temperatures. Expression was normalised against *TraesCS5A02G015600* and the average of *n* = 3 biological replicates is shown for each time point. Variation is shown as ± SE mean (SEM) and is indicated by the shaded region around each point. The light period is indicated by yellow and dark period by grey background shading. ZT0 is the same sample as ZT24 except for D. (a) Expression of *VRN1* under constant 16°C conditions. (b) As for a but for constant 10°C conditions. (c) As for a but for 16°C light/10°C dark conditions. (d) as for a but for 10°C light/16°C dark conditions.

We measured *VRN1* as this is the central regulator of vernalization, and its expression is known to increase under lower ambient temperatures (Yan *et al*., 2003; Dixon *et al*., 2019). We observed a stable level of *VRN1* expression across the constant 10°C time course (Fig. 2a). We then compared this with *VRN1* expression at constant 16°C as this temperature is associated with early season conditions in Northern Europe. Expression showed diurnal control with a peak before dusk (Fig. 2b) and generally higher expression than under constant 10°C. The diurnal expression is reminiscent of that observed in *T. monococcum* in the field, with a peak during the day independent of *VRN2* expression (Nishiura *et al*., 2018).

The expression patterns of constant 10°C and 16°C suggested that the constant 10°C conditions were actually impacting the day expression pattern of *VRN1*; to investigate this, we conducted the 24‐h time course under 16°C by day and 10°C at night DN conditions. Here, we again observed a low, constant level of *VRN1* expression with a slight hint of a predusk increase in expression (Fig. 2c). This indicated that in the facultative spring wheat, it was actually the night temperature that was driving a diurnal expression of *VRN1*. To challenge this, we conducted a final 24‐h time course during which we altered the day and night temperatures to generate an unrealistic 10°C by day and 16°C at night DN time course. Here, the diurnal rhythmicity returned with a peak during the day (Fig. 2d). This highlights that in the facultative spring wheat, *cv*. Cadenza, the expression of the main vernalization gene is regulated by temperature, the effect of which is altered depending on the interaction with the light and dark cycle, most likely via the circadian clock. Furthermore, it highlights a role for warming night temperatures in regulating its expression during the subsequent day.

**Fig. 3:**
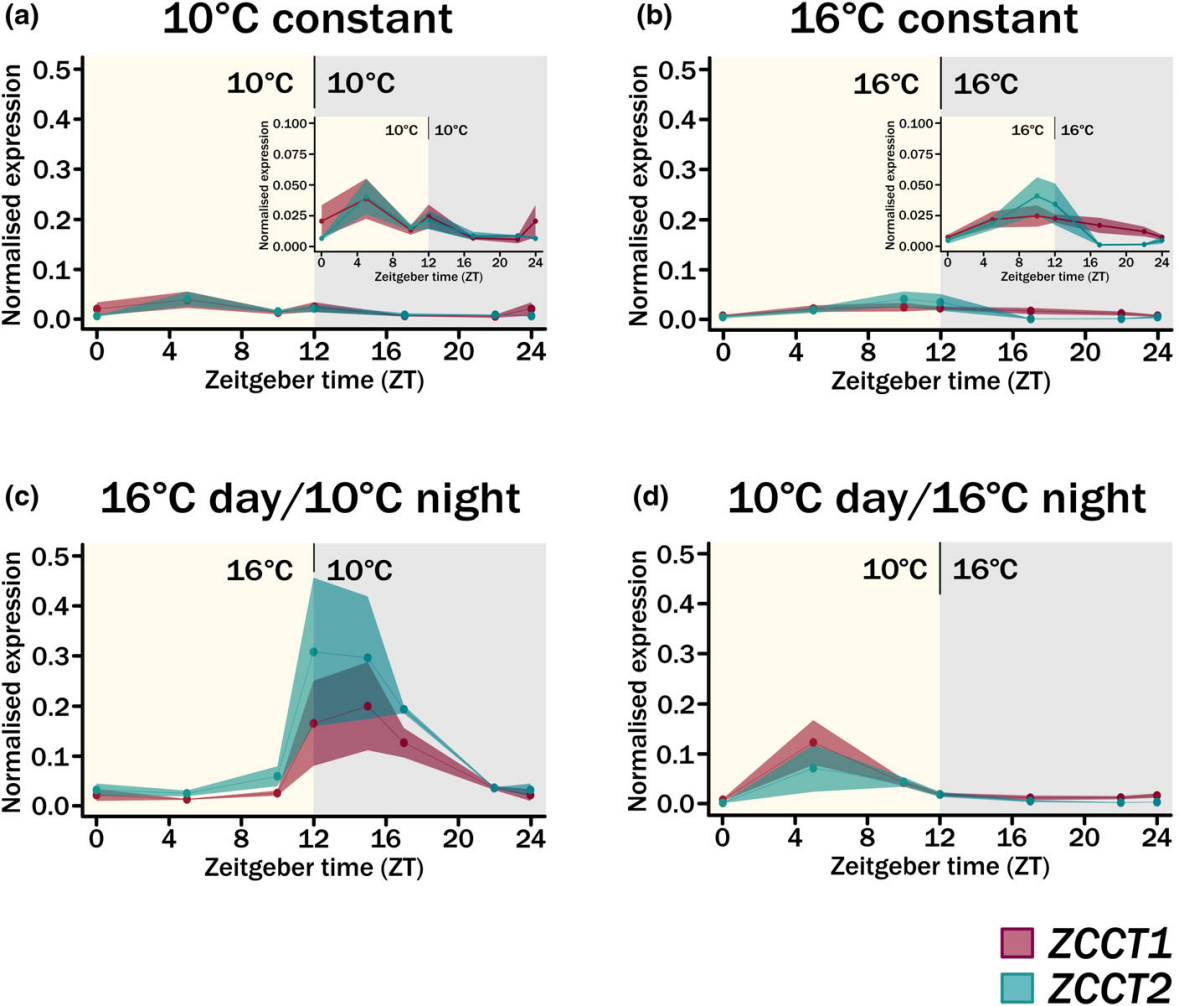
Expression of *ZCCT1* and *ZCCT2* under day‐neutral (DN) conditions with variable temperatures. A ribbon plot showing expression of *ZCCT1* for all three subgenome copies (A, B, D) and *ZCCT2* (A, B, D) in pink and teal respectively in the facultative spring *Triticum aestivum cv*. Cadenza across a 24‐h time course. Leaf tissue was sampled after 3 wk of growth in 12‐h DN conditions under different temperatures. Expression was normalised against *TraesCS5A02G015600* and the average of *n* = 3 biological replicates is shown for each time point. Variation is shown as ± SE mean (SEM) and is indicated by the shaded region around each point. The light period is indicated by yellow and dark period by grey background shading. ZT0 is the same sample as ZT24 except for D. (a) Expression of *ZCCT1* and *ZCCT2* under constant 16°C conditions. (b) As for a but for constant 10°C conditions. (c) As for a but for 16°C light/10°C dark conditions. (d) As for a but for 10°C light/16°C dark conditions.

As a main regulation target of VRN1 is the repression of *VRN2* (Chen & Dubcovsky, 2012), we anticipated that the temperature‐dependent expression patterns observed for *VRN1* would also be reflected through the regulation of *VRN2* under DN photoperiods. Measuring the two *VRN2* genes, *ZCCT‐1* and *‐2*, as homoeologous groups across the previously described 24‐h time courses we observed quite distinct patterns of regulation (Figs 3, S1). Under constant 10°C DN, both *ZCCT‐1* and *‐2* expressions were highly reminiscent of the low, constant expression observed for *VRN1* (Fig. 3a). However, unlike *VRN1* under constant 16°C DN, both *ZCCT‐1* and *‐2* still had a low level of expression (Fig. 3b). Only when considered on a higher resolution scale, due to the low actual expression levels, could *ZCCT‐2* be identified to show a similar diurnal pattern to that observed for *VRN1* (c.f. Figs 2b, 3b) whilst *ZCCT‐1* lacked this rhythmicity. This supported previous reports that the *ZCCT* genes are only expressed under longer photoperiods (Dubcovsky *et al*., 2006; Trevaskis *et al*., 2006). However, when we measured *ZCCT‐1* and *‐2* expression under changing light/dark temperature conditions, both were expressed and showed a diurnal pattern in expression, which was synchronous between the genes (Fig. 3c). Notably, depending on the temperature pattern, the peak in expression shifted such that under warm days, the peak was across and just after dusk whilst with warm nights, it was during the light period. Under these conditions, the expression of *VRN2* is asynchronous with *VRN1*. Furthermore, through comparing the four 24‐h time courses, it identified that temperature is an important regulator in the expression of these genes and that the interaction between the genes may be more complex as the patterns of their expression are altering with variable conditions. We therefore aimed to understand how the vernalization genes expression was responding across a growing season.

### *ZCCT1* expression increases with photoperiod post‐vernalization

**Fig. 4:**
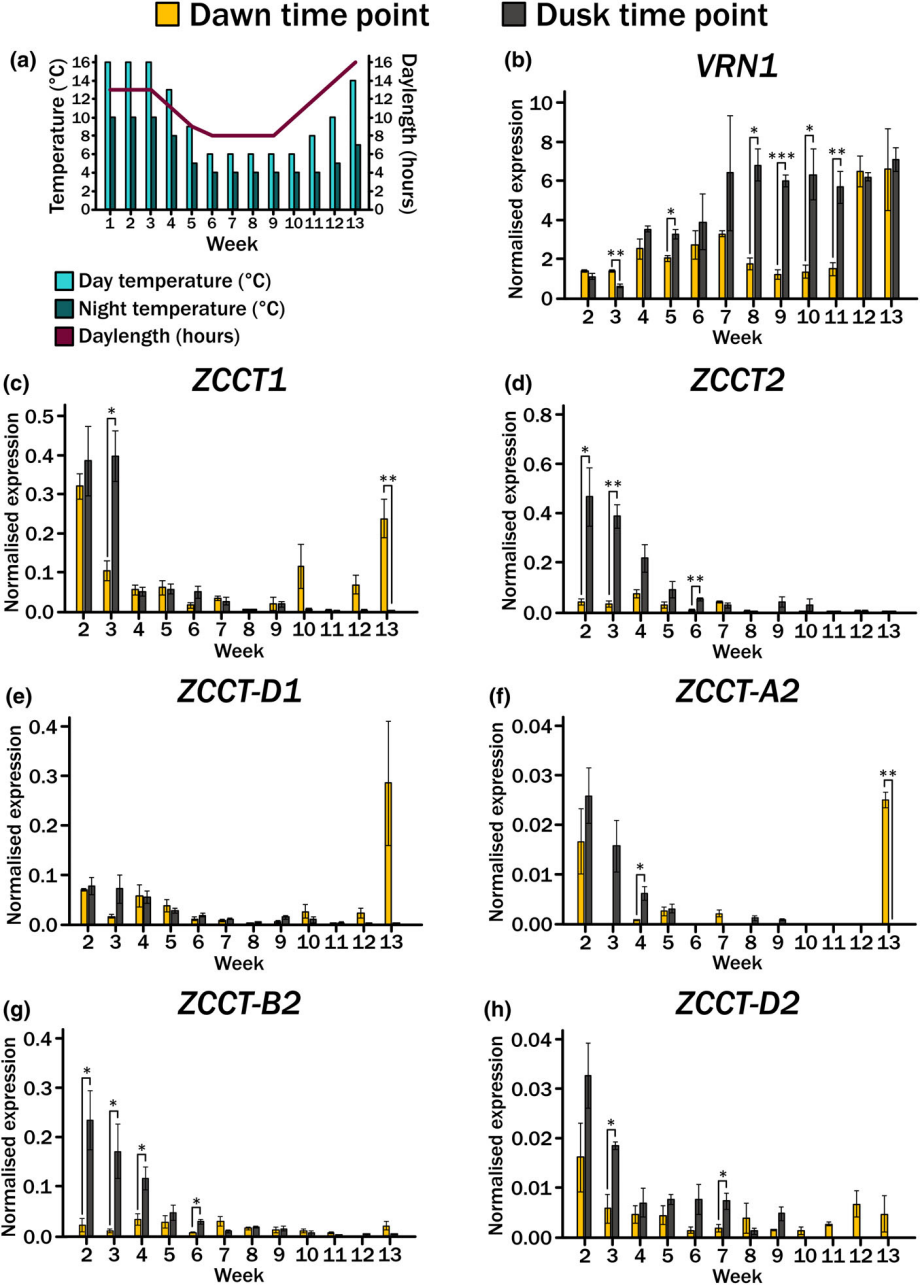
Gene expression across the long‐term seasonal time course at dawn and dusk for the facultative spring wheat *cv*. Cadenza. (a) A graph outlining the temperature conditions for each week during the day (yellow) and night (grey), as well as the daylength indicated by number of hours of light (red). (b) Bar graphs showing gene expression across the long‐term seasonal time course for facultative spring wheat *Triticum aestivum cv*. Cadenza. Leaf tissue was sampled 1 h after the relative ‘dawn’ and ‘dusk’ time points and *n* = 3 for each time point. Expression of *ZCCT1* was normalised against *TraesCS5A02G015600* and ± SE mean (SEM) is indicated by the error bar. *, *P <* 0.05; **, *P* < 0.01; ***, *P* < 0.001 determined by paired Student's *t*‐test only between dawn and dusk timepoints. (c) As for b, but for expression of *ZCCT2*. (d) As for b, but for expression of *VRN1*. (e) As for b, but for expression of *ZCCT‐D1*. (f) As for b, but for expression of *ZCCT‐A2*. (g) As for b, but for expression of *ZCCT‐B2*. (h) As for b, but for expression of *ZCCT‐D2*.

The role of varying day and night temperatures in the regulation of the vernalization genes' expression suggested that both environmental conditions play an important role in the regulation of these genes. It also indicated that even in a facultative spring wheat the role of cold was important for the synchronisation of gene expression. We therefore wanted to test this across a more realistic time course. To do this, we generated an experimental profile in which we took the approximate annual environmental conditions for London, UK. We calculated the average day‐length, temperature during the light‐period and temperature during the dark‐period for each calendar month to generate a time course over 12 weeks with each week containing the average environmental condition for one calendar month. We added in one more week of the mid‐winter condition to ensure that the plants experienced a true vernalization period in case our experimental time course was not sufficient (Fig. 4a). *Cv*. Cadenza seeds were stratified and then grown in soil from Day 0 of Week 1. Under these conditions, *cv*. Cadenza flowered following 125 d ±1.73 SD, which confirmed that the conditions were sufficient to support floral development.

To assess the gene expression, leaf tissue from the newly emerging leaf was sampled each week one hour after dawn and dusk. Confirming the observations from the 24‐h time course (Figs 2 and 3), gene expression of *VRN1* and ‐*2* differed at these two time points. *VRN1* showed a steady increase across the time course, as previously reported (Yan *et al*., 2003; Xie *et al*., 2019), consistent with its role in floral activation (Fig. 4b). However, of possible equal biological interest is the substantial over threefold increase between Weeks 11 to 12 observed at the dawn time point. This coincides with an increasing day temperature from 8°C to 10°C, night temperature from 4°C to 5°C and photoperiod 12 to 14, as well as representing the end of the vernalization period.

The *ZCCT* genes also showed differences in expression between the dawn and dusk time points (Fig. 4c–h). For both *ZCCT‐1* and *‐2*, the dusk time point showed a steady decrease in expression as photoperiod and temperature decreased from Weeks 2 to 5. Expression then remained largely repressed across the time course (Fig. 4c–h); this is consistent with its repression as a result of vernalization (Yan *et al*., 2004). However, like *VRN1*, the same pattern was not observed at the dawn time point. Here, the genes not only showed a different regulation to dawn but also between the two *ZCCT* genes. For *ZCCT‐1*, expression also decreased at dawn with the decreasing temperature and photoperiod, but it was not robustly repressed when temperature and photoperiod started to increase again, at Week 10 onwards (Fig. 4c). This is in contrast to *ZCCT‐2*, which was also sequentially repressed across the time course at the dusk time point as temperature and photoperiod decreased, but was hardly expressed at the dawn time point (Fig. 4d).

As *ZCCT‐1* and *‐2* showed different expression responses across the seasonal time course, we next asked whether these differences extended to the level of homeologous genes. To assess this, we designed gene specific RT‐qPCR primers. Due to the high level of homology between the genes, we were only able to design primers that were specific for each of the *ZCCT‐2* genes and *ZCCT‐D1* (Table S3). Between the *ZCCT‐2* genes, there were distinct expression patterns, with *ZCCT‐A2* and *‐D2* (Fig. 4f,h) showing an increase in expression following the winter‐simulated period whilst *ZCCT‐B2* did not (Fig. 4g). This trend was not observed using the generic *ZCCT‐2* primers, possibly because the expression level of *ZCCT‐A2* and *‐D2* was very low (10‐fold lower). *ZCCT‐D1* expression followed that of the generic primers, although its expression was lower than the generic primers at the start of the time course (Fig. 4e). For all of the genes, there were differences in expression between the dawn and dusk time points.

### Early tiller development is accelerated in *VRN* ‐ *D2* TILLING lines

**Fig. 5:**
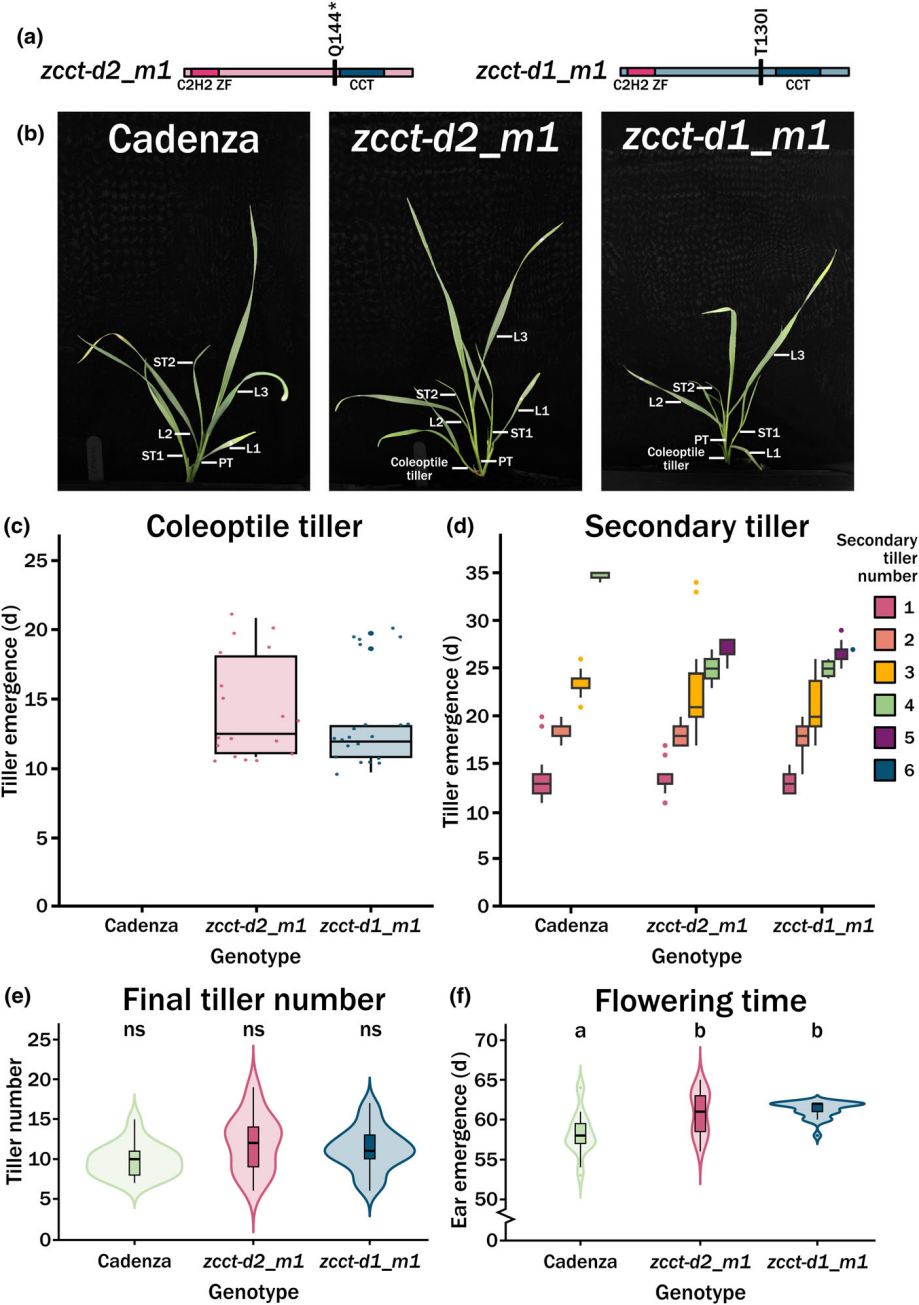
Tiller growth in *VRN2* mutants. (a) An infographic outlining the locations of the mutations in the *Triticum aestivum* TILLING lines used in this study, *zcct‐d2_m1* with a premature termination codon at Q144*, and *zcct‐d1_m1* with a T130I missense mutation. All germplasm used was at the BC_2_F_2_ stage, homozygous for the mutations shown. The relative positions of the C_2_H_2_ zinc finger (pink) and CCT (blue) domains are also as illustrated. (b) A labelled image of example wheat plants for the *cv*. Cadenza control, *zcct‐d2_m1* and *zcct‐d1_m1* with the primary tiller (PT) and coleoptile tillers indicated, along with the emerging leaves (L1, L2, L3) and secondary tillers (ST1, ST2) numbered in ascending order. (c) Box plots showing the median average time (as indicated by the horizontal line) in days until emergence of a new coleoptile tiller in the *cv*. Cadenza control (*n* = 19) compared with the two independent mutant lines; *zcct‐d2_m1* (*n* = 2 1) and *zcct‐d1_m1* (*n* = 24). The box indicates the interquartile range and whiskers indicate the maximum and minimum values excluding outliers. (d) As for c, but for emergence of secondary tillers, with colours indicating the secondary tiller number (1 = pink, 2 = orange, 3 = yellow, 4 = green, 5 = purple, 6 = blue). (e) Violin plots showing the final tiller number, where ns = not significant and a difference in letter (a or b) between the WT and mutant lines indicates *P <* 0.05, as determined by a Kruskal–Wallis test followed by a pairwise Wilcoxon rank sum test with Bonferroni correction. The full range is indicated by the furthest vertical points of the violin, whiskers indicate the 25–75^th^ percentile, box indicates the interquartile range and horizontal line indicates the median. The width of the violin indicates frequency. (f) As for e, but for flowering time defined as half‐ear emergence (GS55).

The expression of the *ZCCT* genes and subgenome copies suggested that in facultative spring wheat, they can function beyond the vernalization response. Therefore, we aimed to identify whether any of the *VRN2* genes controlled important developmental traits without significantly altering the facultative spring vernalization response. To do this, we utilised the Cadenza TILLING collection (Krasileva *et al*., 2017) and were able to identify TILLING mutants, which contained a SNP within the coding sequence of two of the *ZCCT* genes. The line *Cadenza0810* contained a predicted stop Q144* just before the CCT domain in *ZCCT‐D2*, hereafter *zcct‐d2_m1*, whilst *Cadenza1436* contained a T130I, also preceding the CCT domain in *ZCCT‐1D*, hereafter *zcct‐d1_m1* (Fig. 5a).

To reduce the possible impact of background mutations, each line was backcrossed twice and homozygous mutations were selected to generate homozygous BC_2_F_2_ lines. These lines were grown under 16°C long day (LD) conditions to replicate conditions at the start of autumn, and the plants were carefully phenotyped (Fig. 5b). Noticeably, a coleoptile tiller developed in the two TILLING mutants but not *cv*. Cadenza (Fig. 5c). Then, across the first 30 d of growth, both *zcct‐d2_m1* and *zcct‐d1_m1* developed an additional two to three tillers than *cv*. Cadenza (Fig. 5d). This increase in early tiller development was not maintained, and final fertile tiller number was similar between all lines (Fig. 5e). The increase in early tiller development was, however, reflected by a slight but significant (*P* = 0.01) delay in flowering time of 2.5 d ±2.74 SD for *zcct‐d2_m1* and 2.9 d ±1.03 SD for *zcct‐d1_m1* as measured by half spike emergence (Waddingtons GS55; Fig. 5f). This slight delay did not affect the spikelet number (Fig. S2).

### *ZCCT* genes co‐regulate their expression

**Fig. 6:**
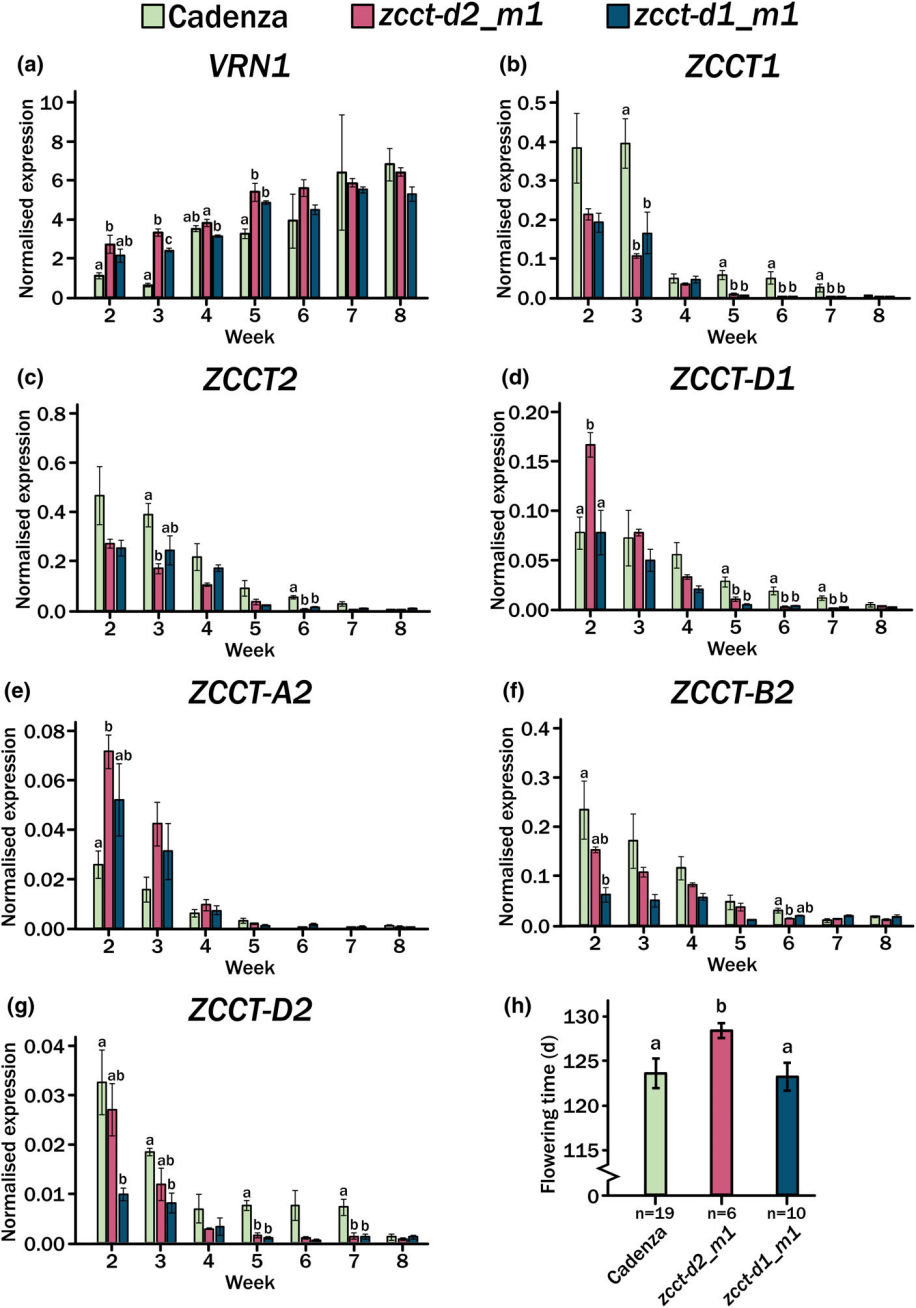
Gene expression across the long‐term seasonal time course for the facultative spring wheat *cv*. Cadenza and mutant lines *zcct‐d2_m1* and *zcct‐d1_m1*. Bar graphs showing gene expression across the long‐term seasonal time course up to Week 8 for facultative spring wheat *Triticum aestivum cv*. Cadenza (green) and mutant lines *zcct‐d2_m1* (pink) and *zcct‐d1_m1* (blue). Leaf tissue was sampled 1 h after the relative ‘dusk’ time point and n = 3 for each time point. Expression was normalised against TraesCS5A02G015600 and ±SE mean (SEM) is indicated by the error bar. Significance was determined by a one‐way ANOVA followed by a Tukey's Honestly Significant Difference *post hoc* test only between wild‐type and mutant lines. A difference in letter (a or b) between the WT and mutant lines for each time point indicates *P <* 0.05, whilst the absence of a letter indicates no significant difference. (a) Expression of *ZCCT1*. (b) Expression of *ZCCT2*. (c) Expression of *VRN1*. (d) Expression of *ZCCT‐D1*. (e) Expression of *ZCCT‐A2*. (f) Expression of *ZCCT‐B2*. (g) Expression of *ZCCT‐D2*. (h) The flowering time for each genotype (*cv*. Cadenza in green, *zcct‐d2_m1* in pink, *zcct‐d1_m1* in blue) following growth in the outlined conditions. Error bars indicate SD; *n* = 19 for *cv*. Cadenza, *n* = 6 for *zcct‐d2_m1* and *n* = 10 for *zcct‐d1_m1*.

As we observed an early‐stage developmental phenotype, we next asked whether this altered the expression of the vernalization genes. To test this, we employed our condensed season time course, as described for Fig. 4a. We sampled emerging leaf tissue at dusk and measured the expression of vernalization genes in *cv*. Cadenza, *zcct‐d2_m1* and *zcct‐d1_m1* (Fig. 6). Here, we observed that in plants carrying mutant alleles of *ZCCT‐D1* or *‐D2*, *VRN1* expression was higher, and this was particularly apparent at the start of the time course (Weeks 1 and 2) (Fig. 6a). This increase in *VRN1* was also reflected by a generally lower expression level of *ZCCT‐1* and *‐2* as measured using the generic primers for these genes (Fig. 6b,c). This supports the molecular inter‐regulation of these genes, as previously described (Yan *et al*., 2004; Chen & Dubcovsky, 2012). Interestingly, this general trend was not maintained when we measured the expression of individual *ZCCT‐1* and *‐2* genes. Here, we observed that the function of the *ZCCT* genes impacted the expression of other *ZCCT* genes. The expression of *ZCCT‐A2* was increased in both lines, which carried either less functional *ZCCT‐D1* or *‐D2*, suggesting that usually these genes repress the expression of *ZCCT‐A2*. Similarly, at the start of the time course, *ZCCT‐D2* expression was much lower in the *zcct‐d1_m1*, suggesting that *‐D2* is activated by *‐D1*. The reverse was observed in the *zcct‐d2_m1*, which had higher expression of *‐D1*, highlighting a possible mutual regulation mechanism (Fig. 6d,g). Significant changes in gene expression were most apparent at the start of the time course, supporting a role for these genes in early developmental regulation. Additionally, we measured flowering times and observed a flowering delay in the *zcct‐d2_m1* line, but not for *zcct‐d1_m1* (Fig. 6h). This indicates that whilst these genes may coregulate the expression of other *ZCCT* genes, this is likely not redundant as the resultant phenotype is variable.

## Discussion

### Vernalization genes are responding to multiple environmental factors

Vernalization is the requirement for cold to enable the vegetative to floral transition and this definition reflects the absolute need for lower ambient temperatures during this process (Dixon *et al*., 2019). Additionally, a seasonal response photoperiod plays an important role, in particular regarding the repression of *VRN2* expression by short‐days (Dubcovsky *et al*., 2006; Trevaskis *et al*., 2006). With the increasingly variable and unpredictable conditions of winter, we aimed to further understand how temperature and photoperiod interacted to regulate the expression of the major vernalization genes in hexaploid bread wheat. We were particularly interested in understanding this function in facultative spring wheat as this growth habit offers a mechanism of increased flexibility to regulate plant development under variable winters, as well as earlier spring sowing which is of interest in more mediterranean climates. We measured *VRN1* and *VRN2* expressions under different temperature patterns with DN photoperiods to further our understanding of how temperature influences the expression of these genes. Interestingly, we observed that both *VRN1* and *VRN2* had altered expression patterns depending on the temperature (Figs 2 and 3). Of particular interest is the altered waveform in expression, which occurred in response to different temperature conditions. This is highly reminiscent of what is observed in barley (*Hordeum vulgare*) and *T. monococcum* (Ford *et al*., 2016; Nishiura *et al*., 2018). It was also notable that the circadian regulation is more robust for *VRN1* under warmer temperatures (Fig. 2b). Furthermore, *VRN2* indicated a possible response to temperature entrainment, such that its expression altered significantly when temperature cycles were introduced and that the phase of the expression altered depending on the cycle temperatures. This is an interesting response, which could hint at possible reasons for altered vernalization responses observed depending on quite small temperature changes, or when major day/night temperature differentials are observed. For example, winters that have greater day/night differences in temperature may take longer for plants to vernalise due to the activation of *VRN2* expression at night. This would be compounded by our observation that *VRN1* expression is greater during the day. Therefore, under certain environmental conditions, *VRN1* and *VRN2* expressions are asynchronous, suggesting other genetic factors are involved in the regulation of these genes.

### Vernalization genes show different responses across development

To better understand how these genes respond under natural conditions, we conducted a seasonal time course. The possibility of multiple factors regulating the vernalization gene expression patterns appears more apparent in the seasonal time course (Fig. 4). Here, *VRN1* expression differs between dawn and dusk time points. At dusk, its expression follows the reported trajectory of increasing during and following vernalization. By contrast, at dawn, expression shows a gradual increase, followed by a decrease and then a sudden increase post‐vernalization as spring conditions are reached. This strongly suggests that the gene is being repressed at dawn until post‐vernalization conditions. Similarly, the *VRN2* genes displayed differential expression patterns between dawn and dusk conditions. Notably, for *ZCCT‐D1* and *‐A2*, expression was not robustly repressed post‐vernalization, suggesting that these genes may have additional roles in regulating wheat development after the floral transition has occurred. These expression patterns strongly imply that the *VRN2* genes have additional roles beyond the timing of the apex transition and that future work dissecting the mechanistic basis of their function in both the vegetative and later stage development would be very interesting. Notably, *VRN1*, in conjunction with *FUL2* and *3*, has an important role in apex patterning and spikelet development (Li *et al*., 2019) and so additional roles for the *VRN2* genes could be anticipated.

### Early tiller growth is regulated by *ZCCT* ‐ *D1* and ‐ *D2*

We aimed to characterise the vernalization genes in a facultative spring wheat and understand whether, through considering the *VRN2* genes separately, we could identify routes to alter winter‐type traits without impacting the floral regulation. To test this hypothesis, we developed two mutant alleles in *ZCCT* genes and phenotyped them under 16°C LD. We observed that the *ZCCT‐D1* and *‐D2* genes were involved in the regulation of lateral branch, or tiller, outgrowth early in plant development (Fig. 5c,d). This increase in tiller outgrowth associated with the presence of *HvVRN2* was also recently identified in barley (Montardit‐Tarda *et al*., 2025). It would be interesting to understand whether this phenotype is based on a common mechanism regarding tiller outgrowth or increases in tiller bud number. Additionally, it would be interesting to understand whether the observed tiller phenotype impacted subsequent leaf development (Shaaf *et al*., 2019). The impact of *VRN2* genes early in development was further supported when we measured the expression of these genes across our condensed growing season time course. Here, we observed differences in gene expression in the first few weeks of the time course, which returned to a similar point by Week 5 (Fig. 6). Given that we observed an early development tiller phenotype, we also phenotyped other growth stages as plant architecture development is often linked (Dixon *et al*., 2020). The final plant phenotypes were extremely mild, with the final tiller number the same between all lines, along with spikelet number (Figs 5e, S1). The absence of major developmental phenotypes at the later growth stages suggests that changes in *ZCCT* gene expression did not significantly impact overall plant growth. However, we did observe a small flowering time change, with the mutant alleles flowering *c*. 2 d later under constant conditions and 5 d later under variable conditions for *zcct‐d2_m1*, which is not the expected result for a floral repressor gene (Figs 5f, 6h). This suggests that the function of the individual *ZCCT* genes differs and that their role in plant development may be quite complex when considered outside the scope of vernalization. It also remains important to further validate phenotypes observed, in particular the regulation of tiller outgrowth, under multilocation field conditions.

### *ZCCT* genes show co‐regulation

Unexpectedly, when we measured the individual gene expression in the mutant backgrounds, we observed that some of the *ZCCT* genes appeared to regulate other *ZCCT* gene expression. For example, between *ZCCT‐D1* and *‐D2*, a regulation loop could be inferred in which a less‐functional *‐D1* caused a reduction in expression of *ZCCT‐D2*, suggesting that normal functional *‐D1* promotes the expression of *‐D2* (Fig. 6g). Whereas the expected less functional *‐D2* led to higher *ZCCT‐D1* expression, suggesting its normal role is to repress the expression of *‐D1*, however, only at the very start of the time course (Fig. 6d). Furthermore, the genetic and expression data also indicate that both *ZCCT‐D1* and *ZCCT‐D2* repress the expression of *ZCCT‐A2* and that this occurred throughout the vernalization period of the time course (Fig. 6e). Therefore, the gene‐specific analysis has identified that the different *ZCCT* genes not only are expressed under the same regulation (Fig. 6) but also are able to regulate their own expression. This is a common regulatory mechanism within adaptive response genes (Rees *et al*., 2022; Gauley *et al*., 2024) but has not been previously reported for cereal vernalization.

**Fig. 7:**
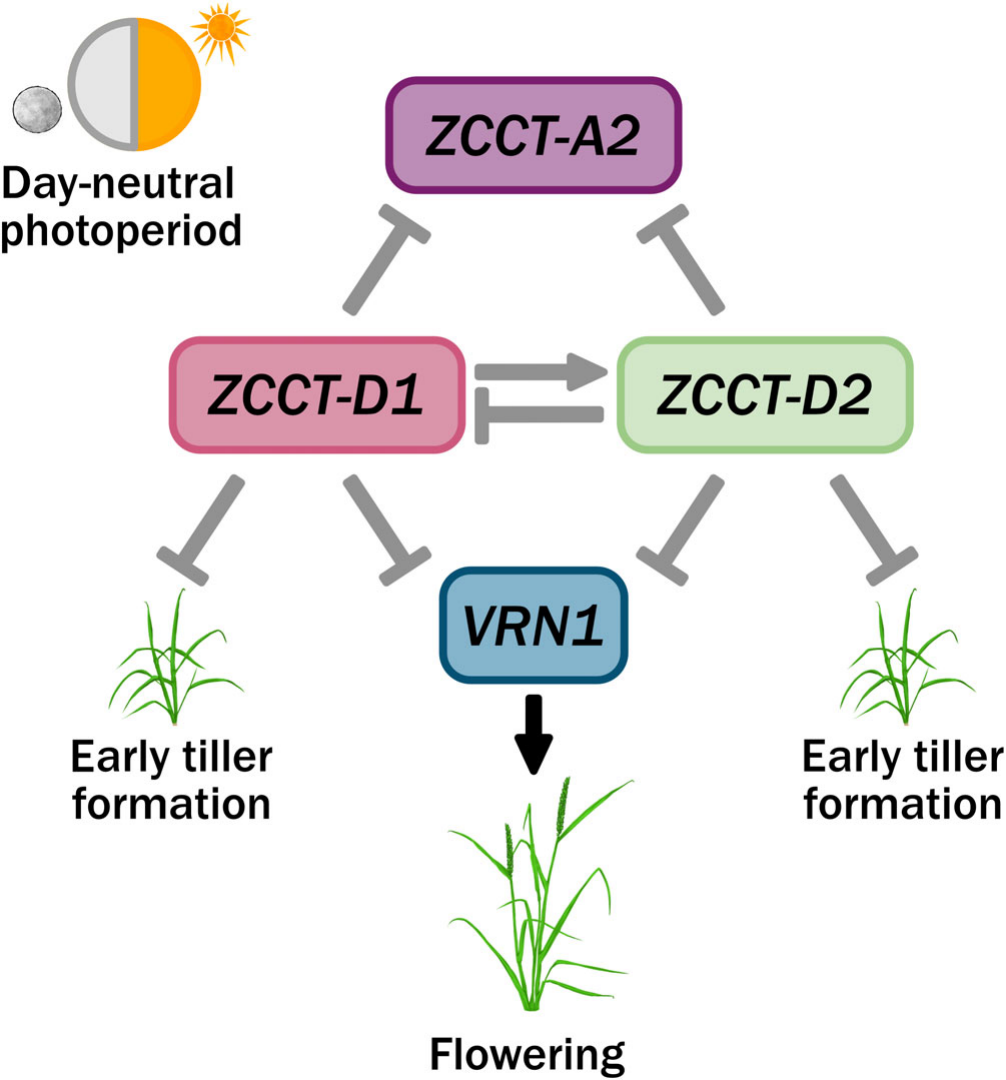
Proposed network outlining interactions between *ZCCT* genes. An infographic outlining a proposed genetic network for the *Triticum aestivum ZCCT* genes from this study in day‐neutral photoperiod conditions. Arrows in grey indicate proposed genetic interactions from the *ZCCT* genes whilst black indicates known interactions. Arrows represent gene activation and bars represent gene repression. *ZCCT‐D1* and *ZCCT‐D2* are proposed to repress *VRN1* and *ZCCT‐A2* expression, as well as early tiller formation through an unknown mechanism. *ZCCT‐D1* is proposed to promote *ZCCT‐D2* expression, which in turn represses *ZCCT‐D1* in a potential negative feedback loop.

Here, we show that the core vernalization genes in facultative spring hexaploid wheat *cv*. Cadenza are regulated differently depending on the temperature conditions experienced and that this regulation causes an asynchronous expression pattern between the main repressor and activator in the vernalization pathway. Furthermore, when considering each of the individual *ZCCT* genes, we identified inter‐regulation in expression as well as independent regulation. Through the development of germplasm, which differed between *ZCCT* genes, we were able to identify variation in early development of tiller growth, which may have applications in the use of facultative spring wheat (Fig. 7). It is a beneficial phenotype due to the increased coverage during early development, which can improve water, light and nutrient efficiency as well as improve competition with biotic stressors (Liang & Richards, 1994; ter Steege *et al*., 2005; Andrew *et al*., 2015). To understand the potential use of these mutants in a seasonal context, they will need to be transferred to current breeding material and tested for early development ground cover in field trials. In particular, it would be of great interest to understand whether the early‐stage tiller development linked with a prostrate phenotype, which has been recorded in wheat and barley (Zhou *et al*., 2018; Marone *et al*., 2020; Kumar *et al*., 2025).

Exploring the roles of specific *ZCCT* genes has shown that these genes have further adaptive potential not only in the context of vernalization but also potentially in other developmental roles. This means these genes may also be involved in regulating development in wheats, which do not have a winter habit, offering the potential to regulate plant development without impacting vernalization *per se*.

## Competing interests

None declared.

## Author contributions

DH and LD conceived, designed and conducted the experiments and analysis. HT, IL and AG designed and conducted experiments. WW supported with bioinformatic analysis. DH and LD wrote the initial manuscript and all authors have confirmed its accuracy.

## Disclaimer

The New Phytologist Foundation remains neutral with regard to jurisdictional claims in maps and in any institutional affiliations.

## Supporting information


**Dataset S1** Promoter analysis of *ZCCT1* and *‐2*.

**Fig. S1** Expression pattern of the individual genome copies of *ZCCT1* and *ZCCT2* under varying temperature conditions in a day‐neutral photoperiod.
**Fig. S2** Final spikelet number for *cv*. Cadenza, *zcct‐d2_m1* and *zcct‐d1_m1* in 16°C LD conditions.
**Table S1** List of germplasm used in this study.
**Table S2** Weekly growth conditions for long‐term gene expression experiment.
**Table S3** List of primers.

**Table S4** Ensembl codes/identified regions for each copy of VRN2 in the 10+ genomes cultivars.
**Table S5** Promoter regions and haplotypes for each copy of VRN2 from wheat pan‐genome.
**Table S6** Orthologous copies of each VRN2 in related grass species.
**Table S7** *VRN1* and *VRN2* alleles in Cadenza.
Please note: Wiley is not responsible for the content or functionality of any Supporting Information supplied by the authors. Any queries (other than missing material) should be directed to the *New Phytologist* Central Office.

## Data Availability

All data are available at https://github.com/TweeticumGEA. Raw data are also provided in File S2. From this study, new germplasm is available following request. Gene identifiers used in this study are from Ensembl v.59 for Chinese Spring v.1.2 for all except *ZCCT‐B2*, which is from *cv*. Julius and are as follows: *ZCCT‐A1* (*TraesCS5A02G541300*); *ZCCT‐B1* (*TraesCS4B02G372700*); *ZCCT‐D1* (*TraesCS4D02G364500*); *ZCCT‐A2* (*TraesCS5A02G541200*); *ZCCT‐B2* (*TraesJUL4B03G02424310*); *ZCCT‐D2* (*TraesCS4D02G364400*); *VRN1* (*TraesCS5A02G391700*, *TraesCS5B02G396600*, *TraesCS5D02G401500*).
