# Supplementary material for: VERNALIZATION2 alters early tiller development in a facultative spring hexaploid bread wheat
Source: New Phytol. 2026 Feb 5;250(6):4022–35. doi: 10.1111/nph.70907 (PMC13193382; doi:10.1111/nph.70907)
Supplement: Supplementary file 2 — Fig. S1 Expression pattern of the individual genome copies of ZCCT1 and ZCCT2 under varying temperature conditions in a day‐neutral photoperiod. Fig. S2 Final spikelet number for cv. Cadenza, zcct‐d2_m1 and zcct‐d1_m1 in 16°C LD conditions. Table S1 List of germplasm used in this study. Table S2 Weekly growth conditions for long‐term gene expression experiment. Table S3 List of primers. [file NPH-250-4022-s002.docx]

## New Phytologist Supporting Information Instructions:

## *New Phytologist* Supporting Information

Article title: *VERNALIZATION 2* alters early tiller development in a facultative spring hexaploid bread wheat

Authors: Dominique Hirsz, Harry Taylor, India Lacey, Wenxue Wu, Adam Gauley & Laura Dixon

Article acceptance date: 04 December 2025

The following Supporting Information is available for this article:


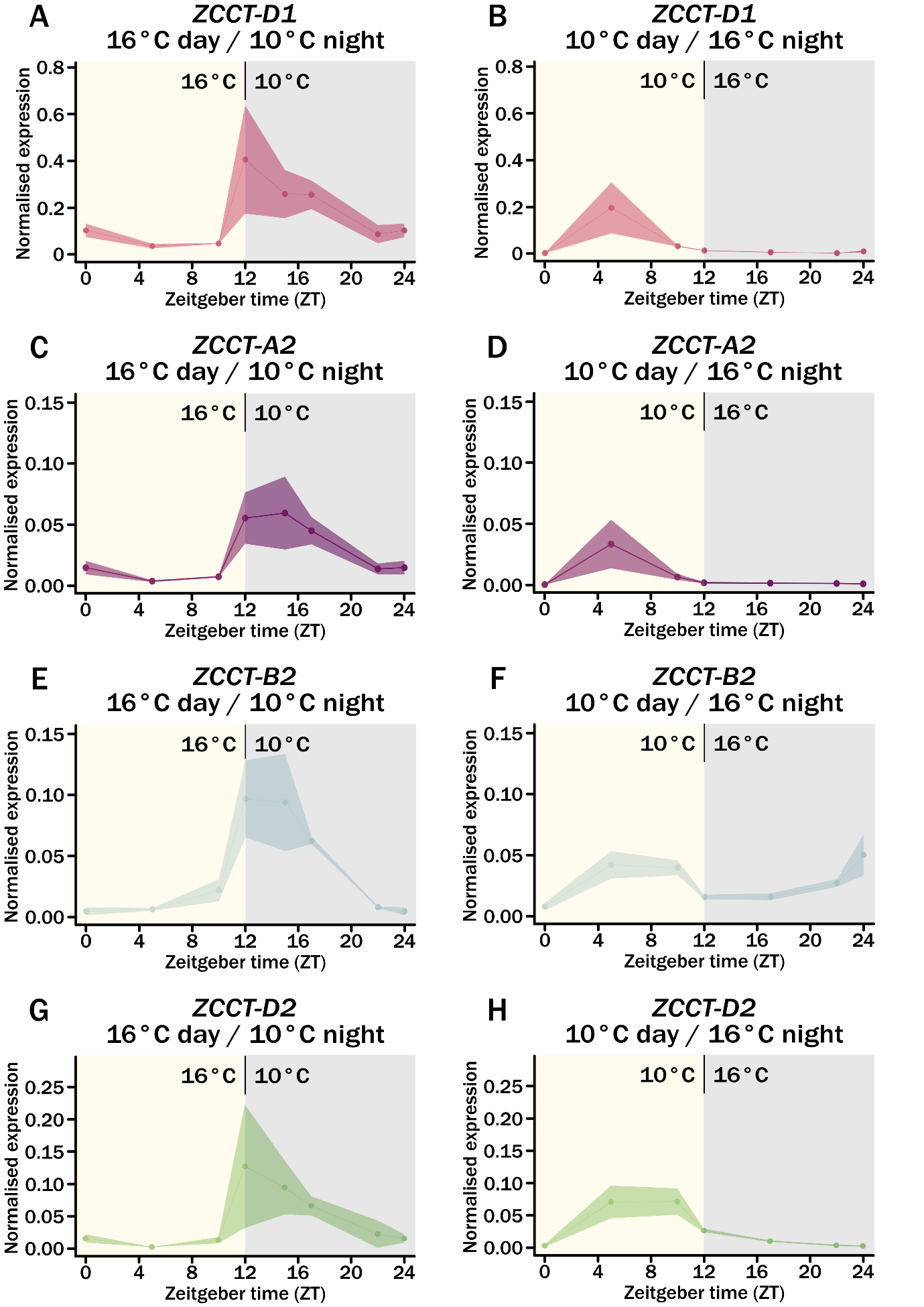


**Fig. S1** Expression pattern of the individual genome copies of *ZCCT1* and *ZCCT2* under varying temperature conditions in a day-neutral photoperiod.

A ribbon plot showing expression of individual copies of *ZCCT1* and *-2* in the facultative spring *Triticum aestivum cv.* Cadenza across two 24-hour time courses. Leaf tissue was sampled after 3 weeks of growth in 12-hour day-neutral conditions under different temperatures. Expression was normalised against *TraesCS5A02G015600* and the average of n = 3 biological replicates is shown for each time point. Variation is shown as +/- standard error mean (SEM) and is indicated by the shaded region around each point. The light period is indicated by yellow and dark period by grey background shading. ZT0 is the same sample as ZT24 for the 16°C light/10°C dark conditions. A) Expression of *ZCCT-D1* under 16°C light/10°C dark conditions, B) as for A but for 10°C light/16°C dark conditions. C) Expression of *ZCCT-A2* under 16°C light/10°C dark conditions, D) as for C but for 10°C light/16°C dark conditions. E) Expression of *ZCCT-B2* under 16°C light/10°C dark conditions. F) as for E but for 10°C light/16°C dark conditions. G) Expression of *ZCCT-D2* under 16°C light/10°C dark conditions, H) as for G but for 10°C light/16°C dark conditions.

**
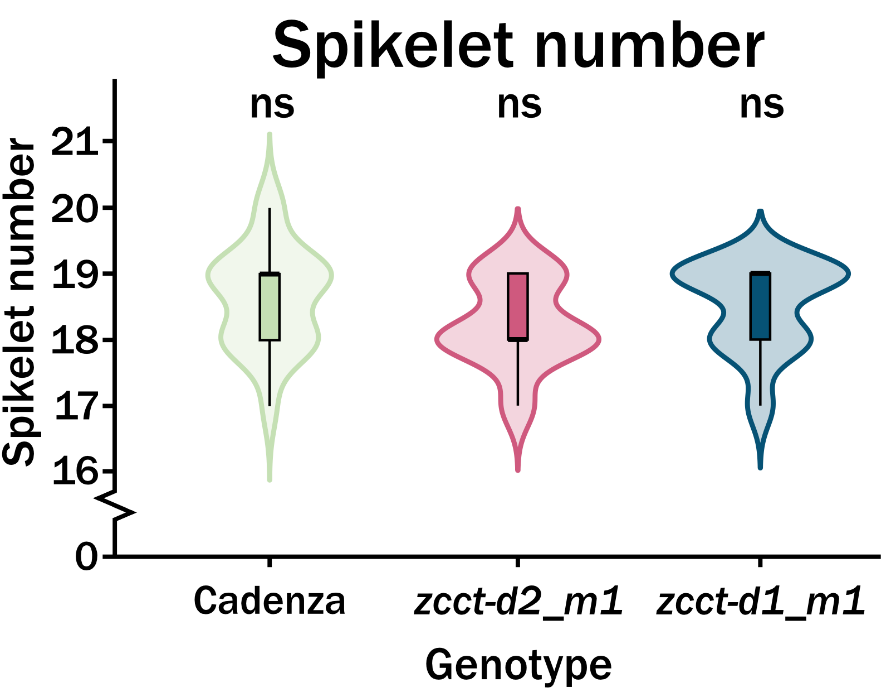
**

**Fig. S2** Final spikelet number for *cv.* Cadenza, *zcct-d2_m1* and *zcct-d1_m1* in 16°C LD conditions.

Total spikelet number was measured for the primary tiller following anthesis in *Triticum aestivum cv.* Cadenza, *zcct-d2_m1* or *zcct-d1_m1*. All spikelets were counted including infertile spikelets. Plants were grown under constant 16°C long day (LD: 16 h light/ 8h dark) conditions. n = 19 plants for Cadenza, n = 21 plants for *zcct-d2_m1* and n = 24 plants for *zcct-d1_m1*; ns = not significant following a Kruskal-Wallis test followed by a pairwise Wilcoxon rank sum test with Bonferroni correction. The full range is indicated by the furthest vertical points of the violin, whiskers indicate the 25-75th percentile, box indicates the interquartile range and horizontal line indicates the median. The width of the violin indicates frequency.

| **Genotype** | **Growth habit** | **Country of origin** | **Background/ Pedigree** |
| --- | --- | --- | --- |
| Cadenza | Facultative spring | UK | Axona / Tonic |
| Cadenza0810 BC2F2 |  | UK | Cadenza x [Cadenza x Cad0810] |
| Cadenza1436 BC2F2 |  | UK | Cadenza x [Cadenza x Cad1436] |

**Table S1** List of germplasm used in this study

All germplasm was hexaploid bread wheat *Triticum aestivum.*

| **Day** | **Week** | **Temperature during day (°C)** | **Temperature during night (°C)** | **Photoperiod (Hours of light period)** | **Sample time 1** | **Sample time 2** |
| --- | --- | --- | --- | --- | --- | --- |
| 1 | 1 | 16 | 10 | 13 |  |  |
| 8 | 2 | 16 | 10 | 13 | ZT1 | ZT14 |
| 15 | 3 | 16 | 10 | 13 | ZT1 | ZT14 |
| 22 | 4 | 13 | 8 | 11 | ZT1 | ZT12 |
| 29 | 5 | 9 | 5 | 9 | ZT1 | ZT10 |
| 36 | 6 | 6 | 4 | 8 | ZT1 | ZT9 |
| 43 | 7 | 6 | 4 | 8 | ZT1 | ZT9 |
| 50 | 8 | 6 | 4 | 8 | ZT1 | ZT9 |
| 57 | 9 | 6 | 4 | 8 | ZT1 | ZT9 |
| 64 | 10 | 6 | 4 | 10 | ZT1 | ZT11 |
| 71 | 11 | 8 | 4 | 12 | ZT1 | ZT13 |
| 78 | 12 | 10 | 5 | 14 | ZT1 | ZT15 |
| 85 | 13 | 14 | 7 | 16 | ZT1 | ZT17 |
| 92 | 14 | 17 | 12 | 16 | ZT1 | ZT17 |
| 99 | 15 | 20 | 14 | 16 | ZT1 | ZT17 |
| 106 | 16 | 20 | 14 | 16 | ZT1 | ZT17 |
| 113 | 17 | 20 | 14 | 16 | ZT1 | ZT17 |
| 120 | 18 | 20 | 14 | 16 | ZT1 | ZT17 |
| 127 | 19 | 20 | 14 | 16 |  |  |
| 134 | 20 | 20 | 14 | 16 |  |  |

**Table S2** Weekly growth conditions for long-term gene expression experiment

Where ZT = zeitgeber time

| **Target gene / SNP** | **Technique** | **Genome target** | **Direction** | **Primer sequence (5’ to 3’)** | **Reference** |
| --- | --- | --- | --- | --- | --- |
| *TraesCS5A02G015600* | RT-qPCR |  | F | TCTAAATGTCCAGGAAGCTGTTA | Borrill et al., 2016 |
|  |  |  | R | CCTGTGGTGCCCAACTATT |  |
| *ZCCT1* | RT-qPCR | All | F | GCAAGAGCCCACATCGTGCC | This study |
|  |  |  | R | GCATTGTGGGATAATGGGCAG |  |
| *ZCCT2* | RT-qPCR | All | F | CACCAGCACTATTAGCAATGCAACG | This study |
|  |  |  | R | CATTGACCCGTGGCCTGAGCTCG |  |
| *ZCCT-D1* | RT-qPCR | D | F | CCAGCTACGCGAGCACCAGTTCTTC | This study |
|  |  |  | R | GTGAGCCTGCTGATGCTGTTCCCTG |  |
| *ZCCT-A2* | RT-qPCR | A | F | AGACGCAGGCGGCCAAAACATG | This study |
|  |  |  | R | CATAGCGGATTTGCTTGTCATAGCACC |  |
| *ZCCT-B2* | RT-qPCR | B | F | CCGGCCAATTGCCACCACTGC | This study |
|  |  |  | R | CAGCGTCCACCTCCAGGGTC |  |
| *ZCCT-D2* | RT-qPCR | D | F | GACGCGGGCGGACAACACAC | This study |
|  |  |  | R | GCCACCATCATCTCTGTATCAATTGTCCTG |  |
| *VRN1* | RT-qPCR | All | F | GAACAAGATCAACCGGCAGGTGAC | Allard et al., 2012 |
|  |  |  | R | GGAGAAGATGATGAGGCCGACCTC |  |
| *ZCCT-D2 Q144* Cadenza0810* | KASP genotyping | D | F | GAAGGTGACCAAGTTCATGCTctgcccataatccgacgatgC | This study |
|  |  |  | F | GAAGGTCGGAGTCAACGGATTctgcccataatccgacgatgT | This study |
|  |  |  | R | GTACCTCATCACCTTCGCCTC | This study |
| *ZCCT-D1 T130I Cadenza1436* | KASP genotyping | D | F | GAAGGTGACCAAGTTCATGCTaggccccaccatcatctttG | This study |
|  |  |  | F | GAAGGTCGGAGTCAACGGATTaggccccaccatcatctttA | This study |
|  |  |  | R | TTTACGGAGGTGCATTCACA | This study |
| *ZCCT-D2 Q144* Cadenza0810* | PCR | D | F | GTACATATCTGTTACCGACAAG | This study |
|  |  |  | R | GATCATAGGGCGAAGTTG | This study |
| *ZCCT-D1 T130I Cadenza1436* | PCR | D | F | GTGCACCTTTGAATGAAAATGG | This study |
|  |  |  | R | GCTGGAGCTCAGCGTAAG | This study |

**Table S3** List of primers

All primers are designed using the Ensembl v.59 *Triticum aestivum* Chinese Spring v1.2 genome, except *ZCCT-B2* which was designed using the *cv.* Julius genome. Gene Identifiers are as follows: *ZCCT1* (TraesCS5A02G541300, TraesCS4B02G372700, TraesCS4D02G364500); *ZCCT2* (TraesCS5A02G541200, TraesJUL4B03G02424310, TraesCS4D02G364400); *ZCCT-D1* (TraesCS4D02G364500); *ZCCT-A2* (TraesCS5A02G541200); *ZCCT-B2* (TraesJUL4B03G02424310); *ZCCT-D2* (TraesCS4D02G364400); *VRN1* (TraesCS5A02G391700, TraesCS5B02G396600, TraesCS5D02G401500). Genome target refers to the three subgenomes (A, B and D) which form hexaploid wheat. Direction refers to the orientation of the primers for PCR F = forward and R = reverse.

| **Gene** | **Allele** | **Associated growth habit** | **Detail** |
| --- | --- | --- | --- |
| ***VRN-A1*** | *Vrn-A1a* | Spring | Promoter insertion |
| ***VRN-B1*** | *vrn-B1* | Winter |  |
| ***VRN-D1*** | *vrn-D1* | Winter |  |
| ***ZCCT-A1*** | *ZCCT-A1a* | Spring |  |
| ***ZCCT-B1*** | *ZCCT-B1b* | Spring |  |
| ***ZCCT-D1*** | Reference | N/A |  |
| ***ZCCT-A2*** | *ZCCT-A2a* | Spring |  |
| ***ZCCT-D2*** | Reference | N/A |  |

**Table S7** *VRN1 and VRN2 alleles in Cadenza*

The genes allele identification follows the standards given in the literature, where Reference is given this is referring to the allelic version found in *Triticum aestivum cv.* Chinese Spring v1.2 ensembl version 59. The associated growth habit is in reference to each allele and not cv. *Cadenza*.

**Dataset S1 supplementary tables 4, 5, and 6**

**Table S4** Ensembl codes/identified regions for each copy of VRN2 in the 10+ genomes cultivars

**Table S5** Promoter regions and haplotypes for each copy of VRN2 from the wheat pan-genome

**Table S6** Orthologous copies of each VRN2 in related grass species

**Dataset S2 Promoter analysis of ZCCT1 and 2**

**References**

Borrill, P., R. Ramirez-Gonzalez, and C. Uauy. 2016. 'expVIP: a Customizable RNA-seq Data Analysis and Visualization Platform', *Plant Physiology*, 170: 2172-86.

Allard, V., Veisz, O., Koszegi, B., Rousset, M., Le Gouis, J. & Martre, P. 2012. ‘The quantitative response of wheat vernalization to environmental variables indicates that vernalization is not a response to cold temperature’. Journal of Experimental Botany, 63**,** 847-57.

Jiao, C., X. Xie, C. Hao, L. Chen, Y. Xie, V. Garg, L. Zhao, Z. Wang, Y. Zhang, T. Li, J. Fu, A. Chitikineni, J. Hou, H. Liu, G. Dwivedi, X. Liu, J. Jia, L. Mao, X. Wang, R. Appels, R. K. Varshney, W. Guo, and X. Zhang. 2025. 'Pan-genome bridges wheat structural variations with habitat and breeding', Nature, 637: 384-93.
